# Performance of nearly fixed offset asymmetric channel-cut crystals for X-ray monochromators

**DOI:** 10.1107/S1600577519011123

**Published:** 2019-10-16

**Authors:** Ronald Frahm, Qianshun Diao, Vadim Murzin, Benjamin Bornmann, Dirk Lützenkirchen-Hecht, Zhen Hong, Tang Li

**Affiliations:** a Department of Physics, University of Wuppertal, Gaußstraße 20, 40227 Wuppertal, Germany; b Institute of High Energy Physics, Yuquan Road 19B, Beijing 100049, People’s Republic of China

**Keywords:** X-ray monochromator, double-crystal monochromators, asymmetric geometry, silicon crystals, QEXAFS, time-resolved spectroscopy, synchrotron instrumentation, X-ray absorption spectroscopy

## Abstract

X-ray double-crystal monochromators face a shift of the exit beam when the Bragg angle and thus the transmitted photon energy changes. This can be compensated for by moving one or both crystals accordingly. In the case of monolithic channel-cut crystals, which exhibit utmost stability, the shift of the monochromated beam is inevitable. Here we report performance tests of novel, asymmetrically cut, channel-cut crystals which reduce the beam movements by more than a factor of 20 relative to the symmetric case over the typical energy range of an EXAFS spectrum at the Cu *K*-edge. In addition, the presented formulas for the beam offset including the asymmetry angle directly indicate the importance of this value, which has been commonly neglected so far in the operation of double-crystal monochromators.

## Abstract 2

A novel, asymmetrically cut, channel-cut crystal was designed and manufactured with the aim to significantly reduce the beam offset change during energy scans. The improvement was verified with synchrotron radiation from an undulator source. The importance of even small, generally so far neglected, asymmetries is pointed out for high-accuracy monochromators.

## 1. Introduction   

A double-crystal monochromator (DCM) is typically equipped with sets of Si(111), Si(220) or Si(311) crystals to obtain monochromatic X-rays especially from synchrotron radiation sources. Commonly, both crystals are mounted together on one goniometer, which rotates both crystals to the desired Bragg angle θ which defines the wavelength λ and thus photon energy *E*. The lattice planes of both crystals have to remain parallel to fractions of an arcsecond. However, if we assume a horizontal axis for the Bragg rotation and a separation between both crystals *D*, the height offset of the exit beam with respect to the incoming white radiation is given by
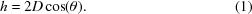
Thus the exit beam continuously moves up to the limiting value 2*D* when the photon energy is increased. Commonly used monochromators maintain a fixed offset by automatically moving one of the crystals (usually the second) vertically to its surface resulting in a change of *D*. In a different setup both crystals are mounted on separate goniometers, and one of them is translated to maintain the desired fixed exit height (Frahm *et al.*, 1995 ▸). Unfortunately, all mechanical movements introduce vibrations, and angular inaccuracies occur. Considering that both crystals have to remain stable in angle to a small fraction of the Darwin width, and with the large distances between monochromators and experiments at high-energy synchrotron radiation sources, such inaccuracies become very difficult to handle. If fast movements are required, stability issues turn out to be more and more of a problem. This is especially obvious when performing quick-scanning EXAFS (QEXAFS) measurements (Frahm, 1988 ▸, 1989 ▸). Here, the monochromator oscillates continuously through a full EXAFS range at nowadays typically up to 50 Hz, yielding 10 ms time resolution (Müller *et al.*, 2015 ▸, 2016 ▸). Whereas for measurements in the seconds range very stable conventional monochromators can be used, for the sub-second range channel-cut crystals made from a monolithic single crystal have been employed, which exhibit ultimate stability. Special crystal shapes can be used to obtain a fixed-offset outgoing beam, but such systems are complicated to fabricate and need demanding alignment procedures (Frahm *et al.*, 2005 ▸). Also, the combination of subsequent Bragg and Laue reflections yields a fixed exit height (Mills, 1983 ▸); however, such a setup has practical drawbacks related to the strongly energy-dependent efficiency of the Laue reflection and the fabrication and stability of the thin Laue crystal.

In channel-cut crystals, the distance of both reflecting crystal surfaces cannot be changed, and equation (1) results in increasing height differences with increasing energy ranges, *e.g.* if several absorption edges have to be covered within one scan. A suitable small slit in the monochromated beam will always illuminate the same sample region when the exit beam of the monochromator, which has to have a larger vertical size, sweeps across the slit. This is important for accurate measurements in X-ray absorption spectroscopy (XAS), especially for powder samples. More information can be found in the publications cited above and in the following. Currently, there seems to be a renaissance of the use of channel-cut crystals for high-stability monochromators, because fewer degrees of freedom improve stability.

The accuracy of the realized crystal orientation with respect to the desired lattice planes specified by different manufacturers increased from about ±0.1° to nowadays ±0.05° or less. Usually, this asymmetry uncertainty is not considered to be a major issue. However, we calculated the changes in exit height of a channel-cut crystal during energy scans for crystals with orientation errors, or if they are intentionally asymmetrically cut. It became obvious that already small orientation errors of ±0.1° had a significant effect on the absolute exit height, and that the asymmetry has to be included in the monochromator control software to maintain an accurate fixed exit height. In the literature a paper by Schildkamp & Meron (1996 ▸) was found about an ‘asymmetric two-bounce monochromator for quasi-fixed offset’, which contained an even more detailed and extended theory, but has been left completely unnoticed during the last decades. The reduction of vertical walk by asymmetric crystals was also discussed, for example, by Hrdý (1989 ▸), Smither & Fernandez (1994 ▸) and Smither *et al.* (2012 ▸), but all without specific experimental verification.

## 2. Theory of asymmetric channel-cut crystals   

**Figure 1:**
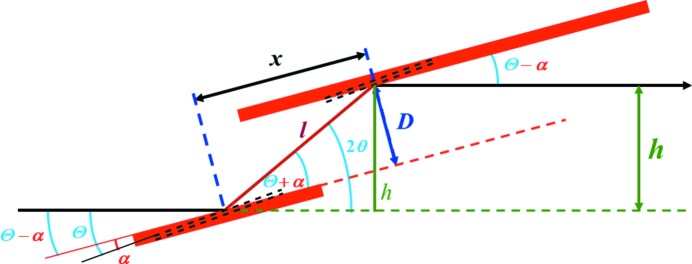
Geometry of an asymmetric channel-cut crystal. The first reflection is defocusing, the second one focuses. The lattice planes of the crystal are indicated by the dashed black lines.

A crystal which is cut with an angle α between its lattice planes and its surface features an asymmetric Bragg reflection, *i.e.* the incoming and outgoing beams have different angles with respect to the crystal surfaces. The general properties of such asymmetric crystals are described in the fundamental publication of Matsushita & Hashizume (1983 ▸) and the review of Caciuffo *et al.* (1987 ▸). The important quantities are shown in Fig. 1 ▸. In the given configuration, the extension of the beam increases after the first reflection, and decreases again after the second. According to Liouville’s theorem, the divergence of the beam between both crystals decreases, and after both reflections the initial beam conditions are restored. In our cases the asymmetry angle is below 1°, and thus the effects on Darwin width and extension of the beam are quite small. However, for larger asymmetries of several degrees, which would be needed for offset optimization at small photon energies, this has to be considered.

**Figure 2:**
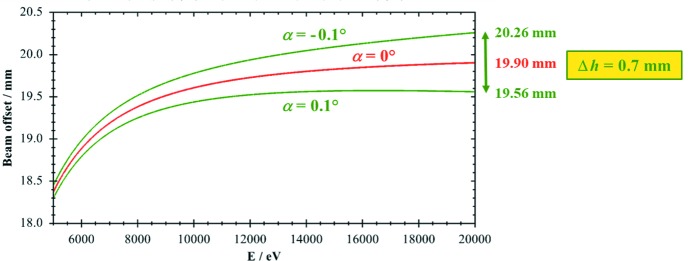
Effect of an asymmetry of ±0.1° on the height of the exit beam. Energy range 5–20 keV, Si(111) crystals, *D* = 10 mm.

For ease of treatment the derivations of the equations in this paragraph follow closely the nomenclature of the paper of Schildkamp & Meron (1996 ▸), and are applicable to all DCMs. By straightforward calculations it follows from Fig. 1 ▸ that the height offset is given by
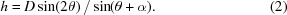
For α = 0 this equation reduces to equation (1) using the equality sin(2θ) = 2sin(θ)cos(θ). The effect of equation (2) on the height offset is calculated for a crystal with a typical gap of *D* = 10 mm, and is shown in Fig. 2 ▸. At the highest energy in the graph of 20 keV the difference due to ±0.1° asymmetry amounts to 0.70 mm, which is not negligible. If the data are extended to 25 keV, the height difference increases to 0.88 mm.

**Figure 3:**
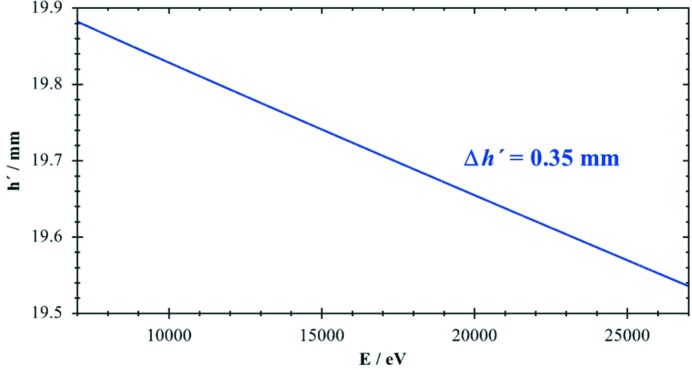
Effect of an asymmetry α = 0.1° on the height *h*′ of the exit beam, see equation (5). Energy range 7–27 keV, Si(111) crystals, *h*_f_ = 20 mm.

To maintain a fixed exit height *h*_f_ in a crystal monochromator with individual crystals the distance *D*(θ) between both must be changed as a function of the Bragg angle according to
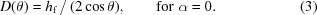
For arbitrary α the general result is given by
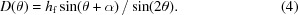
Thus, if the asymmetry is neglected in the monochromator software for the tracking of *D*(θ), the resulting height *h*′ will be incorrect and is given by
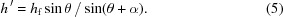
The deviation from the intended fixed exit value *h*_f_ becomes especially important for small Bragg angles (*i.e.* large energies), as shown in Fig. 3 ▸ for a typical value of *h*_f_ = 20 mm and an asymmetry of α = 0.1°. The highest energy of 27 keV includes the Ag *K*-edge, which still can be investigated using Si(111) crystals. The change in *h*′ amounts to 0.35 mm over the full energy range of 20 keV. For a negative asymmetry α = −0.1° the value of *h*′ increases by a similar amount.

Fig. 3 ▸ clearly shows that high-precision operation of a DCM is only possible when asymmetries of the crystals are well known and considered accordingly.

## 3. Asymmetric channel-cut crystals for nearly fixed offset exit beam   

**Figure 4:**
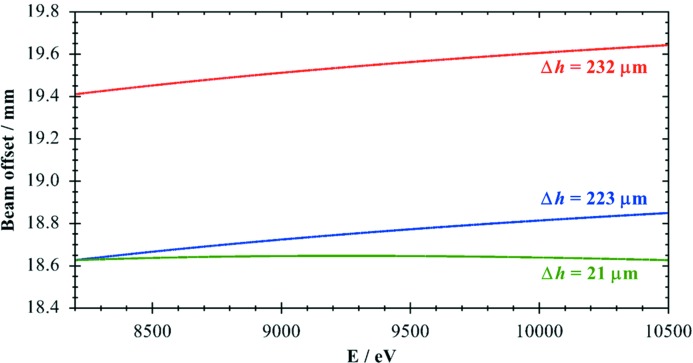
Calculated beam offset for the energy range 8.2–10.5 keV for Si(111) crystals. Red: *D* = 10 mm, α = 0°. Blue: *D* = 9.6 mm, α = 0°. Green: *D* = 10 mm, α_opt_ = 0.6°. Also indicated are the maximum offset height changes Δ*h* in the energy range shown.

Further analysis of equation (2) leads to the result that, for α < 0, the beam offset is continuously increasing with photon energy and larger than in the symmetric case. This also means that asymmetry errors in this direction are especially unfortunate and should be avoided. For α > 0 the offset is always smaller than for the symmetric case, and a local maximum of the offset exists, which is already barely visible in Fig. 2 ▸. This effect can be used to minimize the beam offset in an angular range from θ_1_ to θ_2_ using an optimized α_opt_ by setting *h*(θ_1_) = *h*(θ_2_). This leads to the equation (Schildkamp & Meron, 1996 ▸)
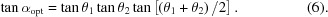
For our experiments we chose to minimize the beam offset change in the energy region 8.2–10.5 keV covering the Ni, Cu and Zn *K*-edges. The results shown in Fig. 4 ▸ demonstrate the strong effect of the asymmetric cut for *D* = 10 mm: the offset changes are reduced by a factor of more than 10 from Δ*h* = 232 µm for a symmetric crystal to Δ*h* = 21 µm. The figure also shows the beam offset for a symmetric crystal with a smaller value of *D* = 9.6 mm, which is calculated from the start offset for the asymmetric case. However, the offset variation is only slightly reduced from the symmetric case for *D* = 10 mm.

**Figure 5:**
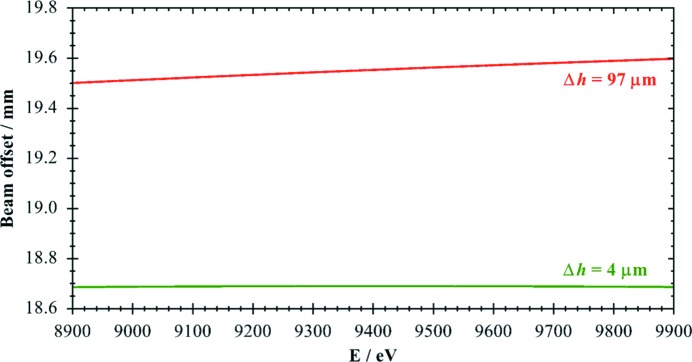
Calculated beam offset for the energy range 8.9–9.9 keV for Si(111) crystals with *D* = 10 mm. Red: α = 0°. Green: α_opt_ = 0.57°. Also indicated are the maximum offset height changes Δ*h* in the energy range shown.

It is impressive to see the improvements for the energy range for an EXAFS scan at the Cu *K*-edge covering 8.9–9.9 keV, as shown in Fig. 5 ▸. Here the offset remains nearly constant within 4 µm for an optimized α_opt_ = 0.57°, and the improvement with respect to a symmetric crystal amounts to a factor of 24.

For the asymmetry α_opt_ = 0.6° used in Fig. 4 ▸ the offset change between 8.9 and 9.9 keV amounts to 6 µm, giving an improvement of 16 with respect to the symmetric case. Those numbers indicate that a very high manufacturing accuracy is needed to fully benefit from the improved performance of asymmetric crystals. If only 200 eV are covered for XANES measurements at the Cu *K*-edge in the range 8950–9150 eV the beam offset can be reduced for *D* = 10 mm at α_opt_ = 0.643° by a factor of about 130 from 21.6 µm to only 171 nm.

**Figure 6:**
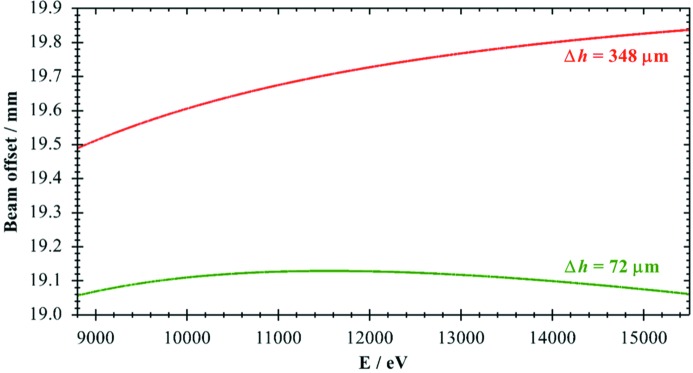
Calculated beam offset changes Δ*h* for the wide energy range 8.8– 15.5 keV for Si(111) crystals with *D* = 10 mm. Red: α = 0°. Green: α_opt_ = 0.30°.

A second asymmetry for the experiments was optimized for the wide energy range 8.8–15.5 keV covering the important Cu and Zn *K*-edges as well as the Pt and Au *L*-edges. As is obvious from Fig. 6 ▸, the reduction in offset change still amounts to a factor of about 5.

## 4. Design of the channel-cut test crystal   

**Figure 7:**
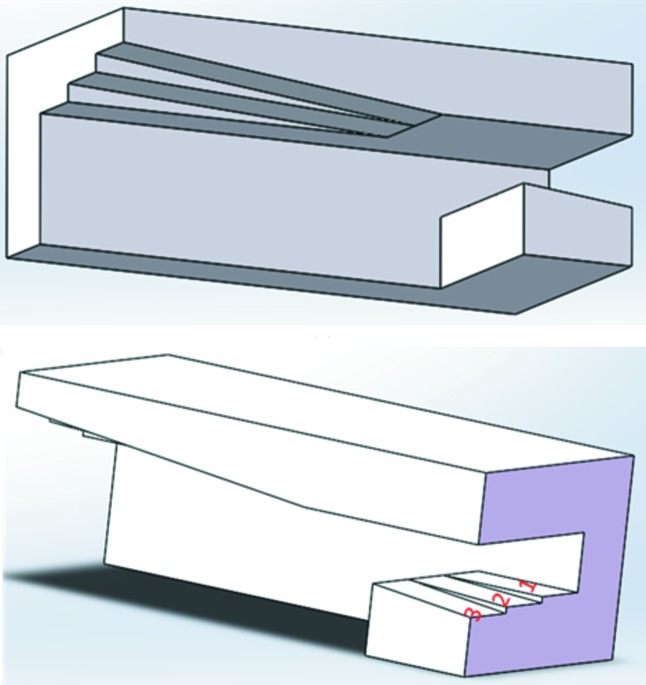
Schematic of the channel-cut test crystal. Surface 1: α_1_ = 0.6°, 2: α_2_ = 0.30°, 3: α_3_ = 0°. For clarity the angles are strongly exaggerated.

To significantly reduce the offset change using asymmetric crystal cuts as described, only plane, parallel surfaces are necessary, which is a tremendous advantage for manufacturing. For the experiments, a symmetric cut, α = 0°, and the two asymmetries described in the previous section with α = 0.30° and 0.60° were designed and realized at the Crystal Laboratory of the Beijing Synchrotron Radiation Facility (BSRF). The design of the Si(111) channel-cut test crystal with all three surface angles is shown in Fig. 7 ▸.

**Figure 8:**
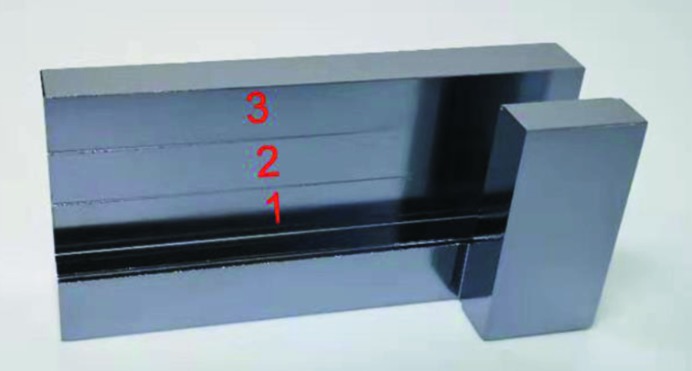
Photograph of the channel-cut test crystal with three strips with the different asymmetries specified in Fig. 7 ▸.

Fig. 8 ▸ shows a photograph of the final crystal for the experiments. The first reflecting surface set is about 21.5 mm long, the second one 95 mm, and the gap was designed to be 10 mm. The crystals are about 12 mm thick, and the connecting back side is 13 mm.

The float-zone silicon crystal rod was grown in China with diameter 130 mm and resistivity greater than 10000 Ω cm, and was used already for a channel-cut crystal with different *D*-values side by side. The asymmetry angles were determined using a crystal cutting chip with Si(111) surface as reference, which was aligned in a diffractometer for crystal orientation. The channel-cut crystal surface was at the same angle as the chip surface. Then the crystal was rotated by angles of 0.3° and 0.6° with an encoder, and the diamond blade saw was adjusted to complete the channel cut. Then the crystal was chemical etched in HF and HNO_3_ solutions.

The asymmetry angles after cutting the channels are reported as α_p_ in Table 2 below. They were measured using a diffractometer using a flat crystal which is separated from the first crystal. It turned out that the asymmetries after production were 0.08° larger than intended. The gap of the asymmetry of the 0.6° cut was measured using a caliper to be 9.9 mm. However, the exact dimensions and final asymmetries were not critical for our investigations – the crystal was only manufactured to test the principles discussed above.

## 5. Experimental setup   

The measurements were performed using monochromatic X-rays using the QEXAFS monochromator (Bornmann *et al.*, 2019 ▸) at beamline P64 (Caliebe *et al.*, 2019 ▸) at the 6 GeV storage ring PETRA III at DESY (Hamburg, Germany). The source of the synchrotron radiation is a 2 m-long undulator, which can be tapered to achieve a large energy band of up to ∼3 keV. The QEXAFS monochromator is equipped with a liquid-nitrogen-cooled symmetric Si(111) channel-cut crystal with a gap of 12 mm to obtain a monochromatic beam. The monochromator was continuously sweeping over a large energy range at up to 10 Hz, yielding 20 flashes of any intermediate energy per second which can be transmitted by the test crystal.

**Figure 9:**
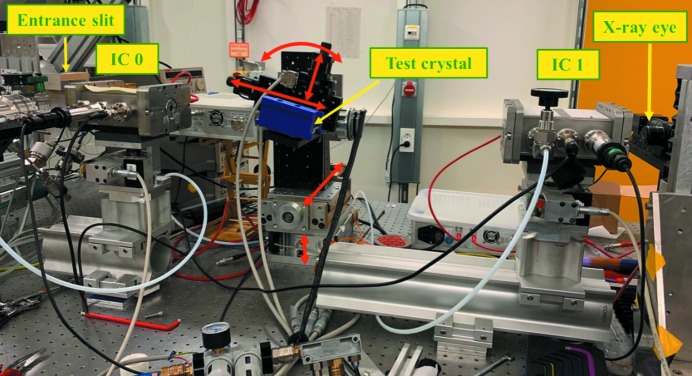
Setup for angular scans and offset determination. The primary beam enters from the left side through the entrance slit. The incoming intensity is detected by ion chamber IC 0, the beam after the test crystal by IC 1 and a 2D X-ray camera. The red arrows show the alignment possibilities for the test crystal, which is mounted inside the blue housing.

The layout of the setup for the offset measurements is shown in Fig. 9 ▸. It was installed in the experimental hutch on a lift table, where usually XAS measurements are performed. The vertically shifting beam from the QEXAFS monochromator was illuminating an entrance slit equipped with Ta blades, which was several millimetres wide, but only 12 µm in height. It defined the radiation source for the experiments.

The channel-cut test crystal was mounted on linear stages on top of a small goniometer defining the Bragg axis, which itself could be aligned with several degrees of freedom. The test crystal was mounted stress-free in a 3D-printed housing. All parts were easily accessible and did not require cooling, which would not be possible if white synchrotron radiation had been used. The continuously oscillating QEXAFS monochromator in the optics hutch allows the test crystal to be rotated to any suitable angle and flashes of the double reflected beam obtained. With a conventional monochromator it would have been necessary to accurately synchronize all angles, whereas in the current setup the test crystal can be arbitrarily rotated within the energy range supplied by the QEXAFS monochromator, and even absorption edges can be measured this way using the test crystal as secondary monochromator. The advantage of using a monochromatic beam for the intended measurements of the offset is also that the zero position of the incoming beam can be measured with accuracy in the micrometre range – this is barely possible when using white radiation, which would also cause heating effects. The beam after the test crystal was measured with ion chambers (Müller *et al.*, 2013 ▸) or a 2D detector with pixel size 7.4 µm × 7.4 µm (‘X-ray eye’; Prosilica GC650M, Allied Vision, Germany). The setup is similar to one used by Ferrari *et al.* (2011 ▸) for the investigation of the intensity of extremely asymmetric channel-cut monochromators.

## 6. Characterization of the test crystal   

The zero angles of the crystal surfaces were determined by moving the test crystal into the beam and by rocking it by small angles. If the centre of the surface is in the rotation axis, a symmetric intensity triangle is expected. The accuracy of this procedure increases with the length of the crystal surface, which is assumed to be perfectly flat. The resulting angular accuracy is estimated to be at least 0.02°.

**Figure 10:**
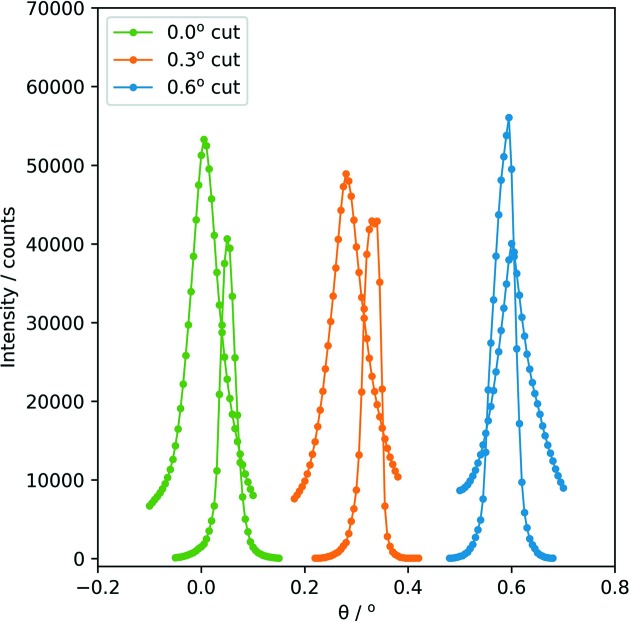
Angular scans of the different crystal cuts. Wider triangles correspond to the short crystal surfaces, the narrower triangles to the longer ones.

We aligned each crystal surface (cuts with 0.0, 0.3 and 0.6°, short and long sides, six surfaces in total) parallel to the X-ray beam and changed the vertical position of the crystal until it blocks exactly half of the beam intensity. Then we scanned the rotation angle of the crystal. By repeating these two types of scans and checking the symmetry of the resulting intensity triangles we were able to align the crystal surface with the rotation axis and find the angle where the crystal is parallel to the beam, as illustrated in Fig. 10 ▸.

The results of the angular and height scans are summarized in Table 1 ▸. The short surface of the 0.6° cut was used as reference, and the position of the angle was defined as 0.6°. The center of rotation was determined only for the short sides. For measurements of the long sides the crystal holder was moved by −50.00 mm along the beam axis.

## 7. Energy scans for calibration of the Bragg angle   

**Figure 11:**
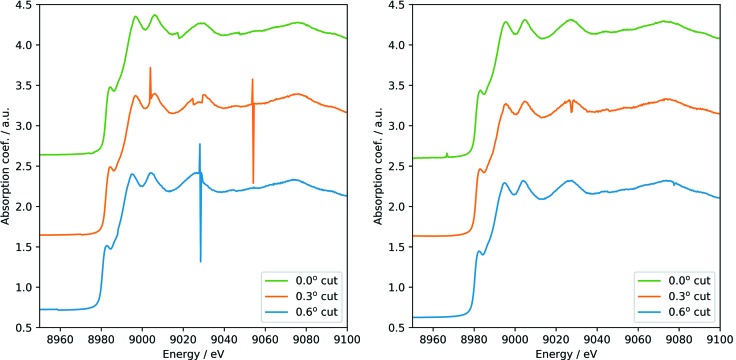
Cu *K*-edge XANES in non-dispersive (left) and dispersive (right) configurations measured with the test crystal for the three strips (see Fig. 7 ▸). Glitches appear when the second channel-cut crystal has another Bragg reflection at a similar angle.

The asymmetry angle can be determined by measuring, for example, a Cu *K*-edge absorption spectrum with the test crystal. The observed angle of the edge, measured with respect to the crystal surface, is shifted by the asymmetry of the test crystal (see Fig. 1 ▸). For the determination of the angle with respect to the Si(111) Bragg lattice plane, the first ion chamber was mounted behind the asymmetric crystal, and an 8 µm-thick Cu foil was placed between both ion chambers. The QEXAFS Si(111) monochromator was oscillating at 10 Hz and 0.6° amplitude in Bragg angle to cover an energy range of about 500 eV at the Cu *K*-edge, yielding just 20 very short flashes per second which were transmitted by the test crystal for each energy value. The angle of the test crystal was scanned with 0.0005° steps, and data were integrated for 5 s for each angle to obtain sufficient statistics. The total time of each scan was 1 h. To change from non-dispersive (+,−,+,−) geometry to the dispersive (+,−,−,+) one, the test crystal was remounted after rotation by 180° around the beam direction, and the measurements were repeated. Both datasets are shown in Fig. 11 ▸. By design of the crystal, the goniometer angles for the different asymmetries are exactly the same. Some distortions in the spectra of the non-dispersive cases were caused by the very difficult beam and intensity conditions for those measurements, which, however, allowed the positions of the Cu *K*-edge to be determined with high precision.

The maximum of the derivative of the XANES spectrum was calibrated to the value of 8980.5 eV (Stümpel *et al.*, 1991 ▸; Kraft *et al.*, 1996 ▸). However, the measured angle with respect to the surface for α = 0° was smaller by 0.06° than expected for the symmetric Bragg case. This indicates that the surface already had a small positive asymmetry with respect to the Si(111) lattice planes, which is qualitatively and quantitatively in good agreement with the measurements after fabrication mentioned above in Section 4. Refraction effects of the X-rays at the crystal surface are smaller than the Darwin width of Si(111) crystals, and thus are so small that they were not considered here. The results clearly demonstrate that the asymmetry angles can be determined from XAS spectra.

## 8. Measurements of the beam offset   

**Figure 12:**
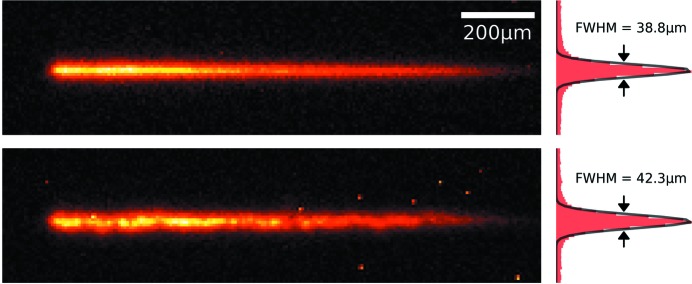
Beam profiles and corresponding normalized histograms with Gaussian fits behind the 12 µm-high slit without test crystal (top) and after double reflection from it (bottom).

The offset changes at different energies for non-dispersive and dispersive channel-cut configurations were directly observed with the 2D X-ray detector as shown in Fig. 12 ▸. The vertical size of the camera sensor is only 3.6 mm, and thus the detector was moved vertically with a linear stage to observe the direct beam from the QEXAFS monochromator behind the 12 µm-high slit and the reflection from the channel-cut test crystal.

The beam profile from each image was fitted with a 2D Gaussian function with an infinite width in the horizontal direction. The offset position was calculated assuming a 7.4 µm vertical pixel size, ideal motion of the vertical stage and a perfect 1:1 optical projection of the beam image on the camera sensor. The vertical beam sizes in Fig. 12 ▸ are larger than the pure slit size mostly due to diffraction by the slit, scattering effects in the fluorescence screen and cross-talk between adjacent pixels due to the high intensity on the detector. The highest intensity is just spread over two pixels amounting to about 15 µm, and the accuracy for measurements of the offset thus is of the order of the slit size. Thus, those effects have no bearing on the conclusions of our study.

**Figure 13:**
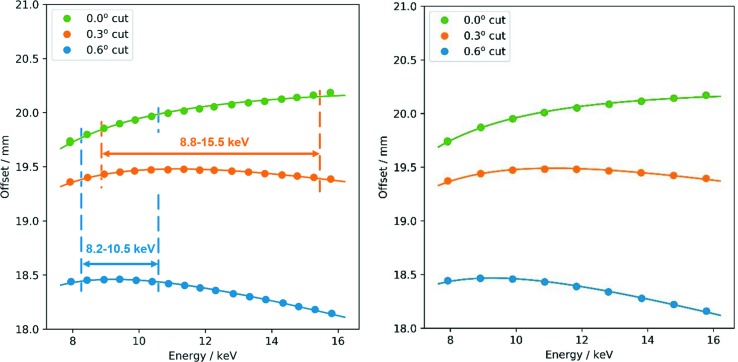
Offset measurements for non-dispersive (left) and dispersive (right) configurations. Experimental points are shown as circles, fits as solid lines. Indicated are the energy ranges for the optimization of the two asymmetries.

The measured offset versus energy is shown in Fig. 13 ▸ together with fits based on equation (2). The asymmetry angle and the gap width were used as fit parameters, and the results are listed in Table 2 ▸.

It is obvious that the offset changes for the asymmetric cuts are strongly reduced with respect to the symmetric case in specific energy ranges, and the experimental data agree excellently with the formulas given above. The offset changes for the energy ranges discussed in Section 3 are also given in Table 2 ▸, calculated using the fitted values for the non-dispersive setup. Due to the very good agreement of the fit results of the non-dispersive and dispersive modes there are also no significant differences in Δ*h* for both cases.

For the large energy range covering 8.8–15.5 keV the vertical offset shift is reduced by a factor of more than three using an asymmetry of 0.3°. In the smaller energy range of 8.2–10.5 keV, covering the Ni, Cu and Zn *K*-edges, the improvement increases to a factor of ten in full agreement with the calculations presented in Fig. 4 ▸. For the energy range 8.9–9.9 keV, which just covers the Cu *K*-edge region (see Fig. 5 ▸), the calculated offset shift amounts to merely 6 µm, a factor of about 15 smaller than in the symmetric case. By using the optimized value of 0.57°, the offset change would be reduced to 4 µm. This clearly indicates that even such a small deviation as 0.031° has a significant effect for high-precision monochromators, and extreme accuracy is needed to fully benefit from the concepts demonstrated here.

## 9. Conclusions and outlook   

We have experimentally demonstrated that the so far inevitable offset shift during energy scans of any double-crystal monochromator, *i.e.* separate crystal or channel-cut designs, could be drastically reduced by using asymmetrically cut crystals, proving the theoretical concepts. If the asymmetry angle is optimized for relatively small energy ranges of 1 keV, covering an EXAFS scan, the offset changes are only of the order of a few micrometres, reduced by more than an order of magnitude with respect to symmetrically cut crystals. The experimental results clearly demonstrate that even small asymmetries of the crystals have significant effects. Due to the fact that the ideal value of α = 0° cannot be realized perfectly, equation (2) should always be considered for accurate control of any double-crystal monochromator, and fitted values as given in Table 2 ▸ should be used for improved performance. In non-focusing setups for XAS measurements, in practice the exit beam of a monochromator usually sweeps across a slit, which defines the beam for the experiment. The part of the intensity which is not transmitted through the slit is lost. It is common not to translate one of the crystals during XAS measurements to keep fixed offset height to avoid vibrations and to maintain highest beam stability. If optimized asymmetrically cut crystals are used, however, a higher intensity will be available for the experiments because the beam stays within the exit slit over large energy ranges, and those slits can be opened up wider. Furthermore, the described improvements are also favorable for XAS experiments of small samples and grazing-incidence experiments.

## Figures and Tables

**Table 1:** Results of the angular and vertical scans for each crystal surface (see text for details)

Cut	Short side, cut angle (^o^)	Long side, cut angle (^o^)	Gap (mm)
0.0°	0.00	0.05	10.2
0.3°	0.28	0.33	10.2
0.6°	0.60	0.60	9.9

**Table 2:** Results of fits of asymmetry angles α and channel gaps *D* to experimental data The offset changes Δ*h* are calculated in the energy ranges given using the fit parameters for the non-dispersive setup. ‘Cut’ refers to the design parameters, α_p_ to the realized values (see Section 4) and α_XAS_ to the results of the XANES measurements.

Cut (°)	* α*_p_ (°)	α_XAS_ (°)	Non-dispersive			Dispersive	
			α (°)	*D* (mm)	Δ*h* (µm)	α (°)	*D* (mm)
0.0°	0.08°	0.06	0.033	10.203	8.8–15.5 keV: 314	0.046	10.222
					8.2–10.5 keV: 223		
0.3°	0.38°	0.36	0.335	10.217	8.8–15.5 keV: 92	0.337	10.225
0.6°	0.68°	0.66	0.601	9.901	8.2–10.5 keV: 20	0.597	9.902
